# Vitamin D receptor, Retinoid X receptor and peroxisome proliferator-activated receptor γ are overexpressed in *BRCA1* mutated breast cancer and predict prognosis

**DOI:** 10.1186/s13046-017-0517-1

**Published:** 2017-04-20

**Authors:** Sabine Heublein, Doris Mayr, Alfons Meindl, Alexandra Kircher, Udo Jeschke, Nina Ditsch

**Affiliations:** 1 Department of Obstetrics and Gynaecology - National Center for Tumor Diseases (NCT), Heidelberg University Hospital, Heidelberg, Germany; 2 Department of Obstetrics and Gynecology, Ludwig-Maximilians-University of Munich, Munich, Germany; 3 Department of Pathology, Ludwig-Maximilians-University of Munich, Munich, Germany; 4 Department of Obstetrics and Gynecology, Technical University of Munich, Munich, Germany; 5 Department of Internal Medicine, SLK-Kliniken Heilbronn GmbH, Heilbronn, Germany

**Keywords:** Vitamin D Receptor (VDR), Retinoid X Receptor (RXR), Peroxisome-proliferator activated Receptor γ (PPARγ), Hereditary breast cancer, Survival, *BRCA1*

## Abstract

**Background:**

*BRCA1* mutated breast cancers are commonly diagnosed as negative for classical hormone receptors i.e. estrogen receptor, progesterone receptor and/or Her2. Due to these common targets being absent the application of anti-endocrine therapies is rather limited and a certain focus has been set on discovering alternative target molecules. We recently highlighted thyroid hormone receptors (TRs) to predict prognosis in breast cancer patients that had been diagnosed a *BRCA1* germline mutation. Vitamin D Receptor (VDR), Retinoid X Receptor (RXR) and Peroxisome Proliferator-activated Receptor γ (PPARγ) are known to interact with TRs by forming functional heterodimers. Whether VDR, RXR or PPARγ are expressed in *BRCA1* mutated breast cancer or may even be present in case of triple negativity is not known. Hence the current study aimed to investigate VDR, RXR and PPARγ in *BRCA1* ^*mut*^ breast cancer and to test whether any of the three may be associated with clinico-pathological criteria including overall survival.

**Methods:**

This study analyzed VDR, RXR and PPARγ by immunohistochemistry in *BRCA1* associated (*n =* 38) and sporadic breast cancer (*n =* 79). Receptors were quantified by applying an established scoring system (IR-score) and were tested for association with clinico-pathological variables.

**Results:**

VDR, RXR and PPARγ were detected in over 90% of triple negative *BRCA1* ^*mut*^ breast cancer and were significantly (VDR: *p <* 0.001, RXR: *p =* 0.010, PPARγ: *p <* 0.001) overexpressed in *BRCA1* mutated as compared to sporadic cancer cases. VDR and RXR positivity predicted prolonged overall survival only in *BRCA1* mutated cases while such association was not observed in sporadic breast cancer.

**Conclusions:**

In conclusion, this is the first study to describe VDR, RXR and PPARγ in *BRCA1* mutated breast cancer. Based on the data presented here these receptors may be hypothesized to potentially evolve as interesting markers or even targets in hereditary breast cancer. However, independent studies are indispensable thus to confirm this hypothesis.

**Electronic supplementary material:**

The online version of this article (doi:10.1186/s13046-017-0517-1) contains supplementary material, which is available to authorized users.

## Background

Immunoreactivity of estrogen receptor (ER), progesterone receptor (PR) or Her2 in breast cancer tissue provides both predictive and prognostic information. Determination of ER, PR and Her2 has evolved to be mandatory during routine clinical management of breast cancer patients. In general, about two third of breast cancer cases are regarded to express at least ER on immunohistochemistry level [1, 2] making them particularly predestined for anti-endocrine treatment options. On the other hand treatment of those breast cancers being negative for ER, PR or Her2 is considered to be rather challenging. At least partly this may also be caused by the fact that due to the lack of hormone receptors anti-endocrine treatments options do not apply. Hence this group of ER negative or even triple negative (i.e. negative for ER, PR and Her2) breast cancers has attracted extensive attention during the last years [3]. While treatment options in Her2 positive breast cancer have notably improved since the approval of Her2 targeted agents [4], clinical management of triple negative breast cancer remains to be rather challenging [5]. Especially those patients that have been identified to carry a germ line mutation in *BRCA1* are diagnosed as negative for ER, PR or Her2 to an unusually high extend [6]. Since the frequent lack of hormone receptors (ER/PR) or Her2 extensively narrows the application of (anti-) endocrine therapies, *BRCA1* associated breast cancers require a specially tailored therapeutic regimen [7]. As a consequence the search for alternative predictive/prognostic biomarkers is on the increase.

Like ER or PR, which have also been referred to as ‘classical steroid hormone receptors’, Vitamin D Receptor (VDR), Retinoid X Receptor (RXR), Peroxisome Proliferator-activated Receptor γ (PPARγ) and Thyroid Hormone Receptors (TRs) are members of the nuclear hormone receptor superfamily. There are several lines of evidence that VDR, RXR or PPARγ may be of relevance in breast cancer tumor-biology. First, TRs are known to assemble with VDR, RXR and PPARγ by forming functional heterodimers. We recently highlighted thyroid hormone receptors (TRs) to be widely expressed in breast cancer tissue deriving from patients diagnosed with a *BRCA1* germline mutation. TRs were of opposing prognostic significance and silencing of TRalpha appeared to diminish viability of *BRCA1* mutated breast cancer cells [8]. Further, polymorphisms in VDR have been demonstrated to be associated with breast cancer risk [9] and both RXR and PPARγ have been demonstrated to comprise anti-cancer cell activity [10–12].

However, neither VDR nor RXR nor PPARγ have been studied in *BRCA1* associated breast cancer so far. As VDR, RXR and PPARγ can be quantified in cancer tissue easily, they - given them being present in *BRCA1* mutated breast cancer cases at all - may evolve as novel alternative biomarkers, especially for hormone receptor negative or even triple negative breast cancer patients.

## Methods

### Aim of the study

Aim of the current study was to investigate whether VDR, RXR and PPARγ in *BRCA1* ^*mut*^ are expressed in breast cancer tissue and whether any of the three may be associated with clinico-pathological criteria including overall survival.

### Patients

One hundred twenty-four patients diagnosed with sporadic (*n =* 86) or *BRCA1* associated cancer (*n =* 38) of the breast were analyzed retrospectively in the current study. Patients had undergone breast cancer surgery at the Department of Obstetrics and Gynecology of the Ludwig-Maximilians-University of Munich, Germany between 1987 and 2009. Following resection breast cancer tissue underwent formalin fixation and paraffin embedding. Formalin fixed paraffin embedded (FFPE) tissue used in this study had been stored under standardized conditions. Thyroid hormone receptor profiling on the same patient panel has already been published [8]. Seven cases were no longer available and hence VDR, RXR and PPARγ staining was only performed in 79 sporadic and 38 *BRCA1* ^*mut*^ samples. Except from correlation analysis (Table 4), calculations were done on this slightly reduce panel on which VDR, RXR and PPARγ data were available (*n =* 117). Breast cancer of non-specific type (NST) was diagnosed in 91 (91/117, 77.8%) of the patients and 62.1% (72/116) were graded as high grade (G3). A significant fraction of the study sample was staged higher than pT1 (*n =* 73, 62.4%) or presented with lymph node (*n =* 62, 56.4%) or distant metastasis (*n =* 49, 46.2%) at time of initial diagnosis. Sufficient information thus to conclude on breast cancer subtypes (Luminal A (*n =* 15), Luminal B (*n =* 14), Her2 positive (*n =* 25), triple negative (*n =* 18)) was available in 72 cases. Mean age (± STDV) of the cohort was 49.8 ± 13.4 years (*BRCA1* associated cases: 41.9 ± 10.8 years; sporadic breast cancer: 53.6 ± 12.9 years). See Table 1 for further details.

**Table 1:** Patient characteristics, whole study panel

[IRS]					*BRCA1* ^mut^									sporadic								
		*BRCA1* ^mut^	sporad.		VDR			RXR			PPARγ			VDR			RXR			PPARγ		
				*p*	<4	≥4	*p*	<4	≥4	*p*	<4	≥4	*p*	<4	≥4	*p*	<4	≥4	*p*	<4	≥4	*p*
suptype	other	8	18	ns	3	5	ns	1	7	ns	4	2	ns	11	6	ns	11	7	ns	16	2	ns
	NST	30	61		4	25		11	19		12	8		40	21		30	31		55	6	
G	G1-2	8	36	0.015	2	5	ns	2	6	ns	4	3	ns	30	6	0.004	17	19	ns	35	1	ns
	G3	29	43		5	24		10	19		12	7		21	21		24	19		36	7	
pT	pT1a-c	21	23	0.008	3	17	ns	4	17	ns	9	5	ns	14	9	ns	9	14	ns	19	4	ns
	pT2-pT4	17	56		4	13		8	9		7	5		37	18		32	24		52	4	
pN	pN0	20	28	ns	2	18	ns	4	16	ns	9	5	ns	16	11	ns	14	14	ns	22	6	0.046
	pN+	15	47		5	10		7	8		6	4		31	16		23	24		45	2	
pM	pM0	18	21	ns	4	13	ns	4	14	ns	5	6	ns	8	12	0.031	8	13	ns	18	3	ns
	pM+	18	49		3	15		8	10		10	4		34	15		27	22		44	5	
ER	negative	27	24	0.002	6	20	ns	8	19	ns	9	9	ns	12	11	ns	15	9	ns	18	6	0.044
	positive	11	40		1	10		4	7		7	1		28	12		20	20		38	2	
PR	negative	27	23	0.001	4	22	ns	8	19	ns	9	9	ns	14	8	ns	15	8	ns	18	5	ns
	positive	11	41		3	8		4	7		7	1		26	15		20	21		38	3	
Her2	negative	22	25	ns	3	18	ns	5	17	ns	9	8	ns	17	7	ns	12	13	ns	22	3	ns
	positive	6	19		1	5		3	3		4	1		9	10		9	10		17	2	
Triple-negative	no	17	51	0.002	4	13	ns	7	10	ns	11	2	0.007	32	19	ns	25	26	ns	48	3	0.012
	yes	12	6		0	11		2	10		2	7		4	1		3	3		3	3	
Ki67	low	10	50	ns	4	5	ns	4	6	ns	6	4	ns	32	18	ns	29	21	ns	45	5	ns
	high	10	20		2	8		4	6		6	3		13	6		9	11		17	3	
age	≤41.5 y	19	16	0.002	3	15	ns	4	15	ns	4	6	ns	8	8	ns	6	10	ns	15	1	ns
	>41.5 y	19	62		4	15		8	11		12	4		42	19		34	28		55	7	

*ns* not significant

### Study design

Patients’ data used within the current study were retrieved from patients’ charts, from the Munich Cancer Registry and by direct contact in a retrospective manner. Benign tumors of the breast or patients diagnosed for *in situ* carcinoma were excluded. The outcome assessed was patients’ overall survival. Overall mean survival of the cohort was 7.31 years (95% CI: 6.24 - 8.38 years) and mean follow up time was 6.82 years (95% CI: 5.90 - 7.75 years). Mean follow up time for all patients still alive at time of analysis was 5.26 years.

### Assay methods

#### Mutation screening

Mutation analysis was described by Fischer et al. [13] and was performed at a German center for *BRCA1* mutation testing (Technical University of Munich, Munich, Germany) according to a standardized protocol. Briefly, high performance liquid chromatography (dHPLC) and sequencing of conspicuous amplicons was employed thus to analyze PCR products comprising all coding exons of *BRCA1*. Alternatively, direct sequencing of all *BRCA1* amplicons was performed. The NCBI (National Center for Biotechnology Information) cDNA sequence U14680.1 (*BRCA1*) served as a reference. In case of a negative sequencing results multiplex ligation-dependent probe amplification (MLPA) was used to screen for deletions or duplications in *BRCA1*. Variants of unknown significance (VUS) characterized as VUS Class III were not considered as mutations.

#### Immunostaining

Immunohistochemistry of VDR, RXR and PPARγ on FFPE sections had been described by our group [14, 15]. In brief, antibodies detecting VDR (mouse anti human VDR, monoclonal, AbD Serotec, Oxford, UK), RXR (mouse anti human RXR, monoclonal, Perseus Proteomics Inc., Tokyo, Japan) and PPARγ (rabbit anti human RXR, polyclonal, Abcam, Cambridge, MA, USA) were stained by employing standardized procedures and commercially available kits (Vectastain Elite mouse-IgG-Kit for VDR, RXR staining; ZytoChem Plus HRP Polymer System (Mouse/Rabbit) for detection of PPARγ). Placenta tissue which had been demonstrated to express VDR, RXR as well as PPARγ served as positive control [14]. Placenta and breast cancer sections treated with pre-immune rabbit IgG (supersensitive rabbit negative control, BioGenex, Fremont, CA) or isotype matched mouse IgGs (Dako, Hamburg, D) instead of the primary antibody were used as negative controls. Positive and negative controls were included in each experiment. A well-established semiquantitaive scoring system (IR-score or Remmele score) was employed thus to quantify immunostaining in a semi-quantitative manner [14, 16–18]. Scoring was performed by two independent observers by consensus. This scoring method has already been proven suitable to assess VDR, RXR and PPARγ immunostaining [15–17]. The IR-score quantifies immunoreactivity by multiplication of staining intensity (graded as 0: none, 1: weak, 2: moderate and 3: strong staining) and percentage of positively stained cells (0: no staining, 1: ≤ 10% of the cells, 2: 11–50% of the cells, 3: 51–80% of the cells and 4: ≥ 81% of the cells). Tissue samples that had been assigned an IR-Score higher or equal to IRS 4 (i.e. IRS 4, IRS 6, IRS 8, IRS 9, IRS 12) were scored as positive. This cut-off was set based on mean expression of VDR (mean IRS [*n =* 117] = 4.14) and RXR (mean IRS [*n =* 117] = 4.28).

Assessment of Her2 has been described in [19]. Her2 staining of cases that had been diagnosed before routine HER2 staining was performed, was done on archived FFPE samples where available. Thus to conclude on breast cancer subtypes, we also performed Ki67 staining. Samples were stained using an anti-Ki67 monoclonal antibody (Dako, Hamburg, Germany) at a dilution of 1:150 on a VENTANA®-Benchmark Unit (Roche, Mannheim, Germany). Scoring was performed according to our local standards also applying for routine clinical diagnostics: score 0 = 0% positive cells, score 1: < 5% positive cells, score 2: < 10% positive cells, score 3: < 20% positive cells, score 4: > 20% positive cells). Tissue samples that had been assigned a Ki67-Score higher than score 2 (i.e. score 3 or 4) were scored as positive. Overexpression of Ki67 was defined as Ki67-Score 4.

### Statistical analysis methods

This study has been carried out according to the REMARK (Reporting Recommendations for Tumor Marker Prognostic Studies) criteria [20]. The IBM statistic package SPSS (version 23) was used to test data for statistical significance. Fisher’s exact test and the Mann–Whitney test were used thus to test differences for statistical significance. Survival times were compared by Kaplan-Meier analysis and differences in patient overall survival times were tested for significance by using the chi-square statistics of the log rank test.

Statistical analysis were also done in a group of 54 patients (n (sporadic) = 27, n (*BRCA1* associated) = 27). These patients had been matched (*p =* 1.000) according to tumor size, lymph node status, presence of metastasis and tumor grade.

## Results

### Study cohort

There was no significant difference in terms of histologic subtype, presence of lymph node or distant metastasis or Her2 positivity when sporadic cancer cases and those carrying a *BRCA1* germline mutation were compared. *BRCA1* ^*mut*^ cases were smaller in size (*p =* 0.008), less well differentiated (*p =* 0.015) and more often classified as negative for ‘classical’ hormone receptors (ER: *p =* 0.002; PR: *p =* 0.001) as compared to sporadic cancers. In line with this, *BRCA1* ^*mut*^ cancers were more often found to be triple negative (*p =* 0.002). Finally, *BRCA1* ^*mut*^ patients were significantly younger (*p =* 0.002) at the time of primary diagnosis (Table 1).

### VDR, RXR and PPARγ are overexpressed in *BRCA1*^*mut*^ breast cancer cases

**Fig. 1:**
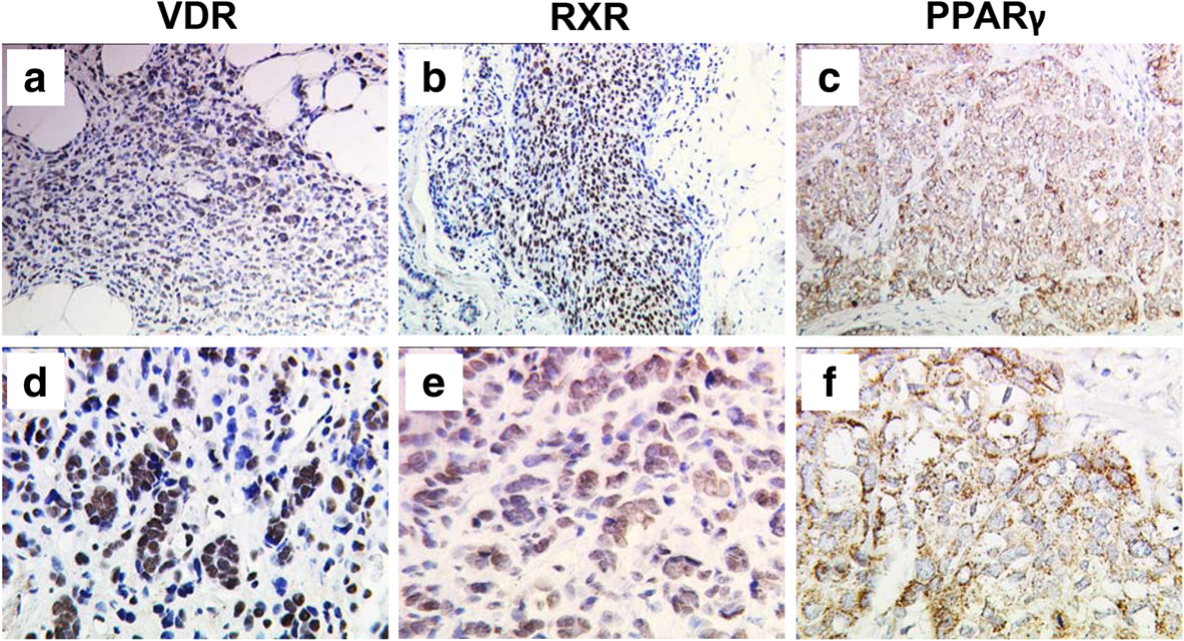
VDR, RXR and PPARγ immunostaining in *BRCA1* mutated breast cancer. VDR, RXR and PPARγ were detected by immunohistochemistry in BRCA1 mutated breast cancer. Representative images are shown; magnification: **a**-**c**: 10 × lens, **d**-**f**: 25 × lens

**Fig. 2:**
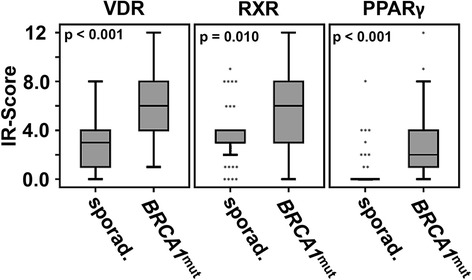
VDR, RXR and PPARγ are significantly up-regulated in *BRCA1* mutated breast cancer tissue. VDR, RXR and PPARγ immunoreactivity of sporadic vs. BRCA1 mutated breast cancer tissue was compared. All the three receptors were found to be significantly up-regulated in a *BRCA1* mutated genetic background. *P*-values derived from relevant Mann–Whitney-U tests are shown

VDR and RXR were expressed with a prominent nuclear pattern, while PPARγ staining was mainly located in the cytoplasm (Fig. 1). In case of all the three antigens staining was found restricted to cancer cells whereas stroma tissue and intercellular spaces stained negative. Regarding the total patient sample both VDR and RXR were expressed in about half of all cases (VDR: 57/115, 49.6%; RXR: 64/117, 54.7%) while much less cases were detected to stain positive for PPARγ (PPARγ: 18/105, 17.1%). Apart from PPARγ overexpression in triple negative cancers (*p =* 0.007) no correlation of clinico-pathological parameters and VDR, RXR or PPARγ positivity was detected in *BRCA1* ^*mut*^ patients (Table 1). In terms of sporadic cancer VDR was overexpressed in poorly differentiated (*p =* 0.004) or non-metastasized (M0, *p =* 0.031) cases. PPARγ was negatively correlated with lymph node involvement (*p =* 0.046) and ER staining (*p =* 0.044). Interestingly, PPARγ (*p =* 0.012) was expressed in half of those sporadic cases that were diagnosed as triple negative. No association of RXR and clinico-pathological variables was detected in terms of sporadic breast cancer. Patients carrying a *BRCA1* germline mutation overexpressed VDR (*BRCA1* ^*mut*^ vs. sporadic: IRS 6.00 vs. IRS 3.00%, *p <* 0.001), RXR (*BRCA1* ^*mut*^ vs. sporadic: IRS 6.00 vs. IRS 3.00, *p =* 0.010) and PPARγ (*BRCA1* ^*mut*^ vs. sporadic: IRS 2.00 vs. IRS 0.00, *p <* 0.001) when compared to sporadic breast cancer (Fig. 2). A similar effect could be reproduced when sporadic and *BRCA1* ^*mut*^ cancers were compared with respect to breast cancer subtypes (Table 2).

**Table 2:** VDR, RXR and PPARγ immunopositivity in breast cancer subgroups

	VDR (median IRS)			RXR (median IRS)			PPARγ (median IRS)		
	*BRCA1* ^*mut*^	sporadic	*p*	*BRCA1* ^*mut*^	sporadic	*p*	*BRCA1* ^*mut*^	sporadic	*p*
Luminal A	3,5	1,0	0,142	6,0	4,0	0,223	1,5	0,0	0,002
Luminal B	8,0	3,5	0,026	6,0	3,5	0,23	1,0	0,0	0,032
Her2 positive	8,0	4,0	0,023	4,5	4,0	0,769	1,0	0,0	0,061
TNBC	8,0	3,0	0,003	8,0	2,5	0,036	6,0	2,0	0,072

Median VDR, RXR and PPARγ immunopositivity was analyzed in breast cancer subtypes.

*BRCA1* mutation has been associated with the basal like breast cancer subtype [21]. Among other criteria loss of hormone receptors, loss of Her2, positivity for Ki67 and poor differentiation have been identified to be characteristics of basal like breast cancers [22, 23]. Our patient sample contained 18 TNBC cases with 14 of them being classified as high grade. Information on Ki67 was available in 13 of 18 TNBC cases. Information on both ki67 and Grading was available in 13 cases. Seven of these 13 TNBC samples were identified as both high grade and overexpressing Ki67, therefore comprising at least ‘basic basal like’ features. Interestingly, 5 out of 7 (71%) *BRCA1* ^*mut*^ cases but only 2 out of 6 (33%) sporadic TNBC cases showed highly proliferative i.e. ‘basic basal like’ characteristics as explained above (Additional file 1: Figure S1). VDR, RXR and PPARγ were expressed in 5 (VDR), 4 (RXR) and 3 (PPARγ) out of 5 highly proliferative *BRCA1* ^*mut*^ TNBC cases.

**Fig. 3:**
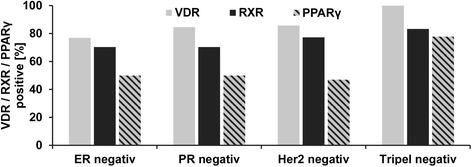
Percentages of VDR, RXR and PPARγ cases in respect to hormone receptor expression. VDR, RXR and PPARγ positive cases in ER negative, PR negative, Her2 negative and triple negative cancer were counted and are displayed as percentage in respect to the respective hormone receptor profile. Even in absence of classical hormone receptors VDR, RXR or PPARγ were detected in the large majority of cases

*BRCA1* ^*mut*^ breast cancer is commonly found to be negative for classical hormone receptors i.e. ER, PR and/or Her2. We then analyzed expression of VDR, RXR and PPARγ in those *BRCA1* ^*mut*^ cases that stained negative for classical hormone receptors. Interestingly, the vast majority of hormone receptor negative, *BRCA1* ^*mut*^ cases were positive for VDR, RXR or PPARγ. In particular, triple negative breast cancer stained positive in up to 100% of the cases (Fig. 3).

Comparisons were repeated in the matched patient panel (Table 3) and overexpression of nuclear receptors in *BRCA1* ^*mut*^ as compared to sporadic cancer cases could be reproduced in terms of VDR (*BRCA1* ^*mut*^ vs. sporadic: IRS 6.00 vs. IRS 3.50 *p =* 0.008) as well as PPARγ (*BRCA1* ^*mut*^ vs. sporadic: IRS 6.00 vs. IRS 4.00, *p =* 0.008). Further, PPARγ remained to be overexpressed in triple negative cancers regarding both *BRCA1* ^*mut*^ (*p =* 0.036) and sporadic (*p =* 0.040) cases. Further, PPARγ was positively correlated to pN0 (*p =* 0.047) in sporadic cancers. A positive correlation of RXR and PR (*p =* 0.008) was observed. Finally, RXR and VDR were found to be inversely correlated to grading (RXR - grading: *p =* 0.022) or presence of distant metastasis (VDR - pM: *p =* 0.041).

**Table 3:** Patient characteristics, matched groups

[IRS]		*BRCA1* ^mut^									sporadic								
		VDR			RXR			PPARγ			VDR			RXR			PPARγ		
		<4	≥4	*p*	<4	≥4	*p*	<4	≥4	*p*	<4	≥4	*p*	<4	≥4	*p*	<4	≥4	*p*
suptype	other	3	2	ns	1	4	ns	4	0	ns	2	3	ns	3	3	ns	5	1	ns
	NST	4	18		9	13		9	7		11	10		8	13		17	4	
G	G1-2	2	5	ns	2	5	ns	4	2	ns	5	2	ns	0	7	0.022	7	0	ns
	G3	5	15		8	12		9	5		8	11		11	9		15	5	
pT	pT1a-c	3	9	ns	3	9	ns	8	2	ns	4	8	ns	5	7	ns	9	3	ns
	pT2-pT4	4	11		7	8		5	5		9	5		6	9		13	2	
pN	pN0	2	13	ns	3	12	ns	8	4	ns	6	8	ns	7	8	ns	10	5	0.047
	pN+	5	7		7	5		5	3		7	5		4	8		12	0	
pM	pM0	4	7	ns	3	8	ns	4	3	ns	2	8	0.041	3	8	ns	10	1	ns
	pM+	3	13		7	9		9	4		11	5		8	8		12	4	
ER	negative	6	13	ns	7	12	ns	8	6	ns	8	8	ns	9	8	ns	12	5	ns
	positive	1	7		3	5		5	1		5	5		2	8		10	0	
PR	negative	4	16	ns	7	13	ns	8	6	ns	9	6	ns	10	6	0.008	12	4	ns
	positive	3	4		3	4		5	1		4	7		1	10		10	1	
Her2	negative	3	13	ns	4	12	ns	8	5	ns	7	4	ns	4	8	ns	9	3	ns
	positive	1	3		2	2		2	1		2	7		2	7		8	1	
Triple-negative	no	4	9	ns	6	7	ns	9	2	0.036	7	10	ns	4	13	ns	16	1	0.040
	yes	0	8		1	7		1	4		4	1		3	3		3	3	
Ki67	low	5	5	ns	6	4	ns	7	3	ns	8	9	ns	8	9	ns	13	4	ns
	high	1	4		1	4		3	1		4	2		3	4		6	1	
age	≤41.5 y	3	9	ns	4	8	ns	3	4	ns	1	3	ns	1	3	ns	4	0	ns
	>41.5 y	4	11		6	9		10	3		12	10		10	13		18	5	

*ns* not significant

### Correlation analysis

Nuclear hormone receptors are known to act as heterodimers. Thus to conclude on potential heterodimerization correlation analysis was performed. Several correlations among VDR, RXR and PPARγ among each other were observed both in *BRCA1* ^*mut*^ and sporadic cases (Table 4). Interestingly, RXR (*BRCA1* ^*mut*^: *p =* 0.014) and PPARγ (sporadic: *p =* 0.035) immunoreactivity was found to rise in parallel to TRβ positivity, while no correlation to oncogenic TRα was observed.

**Table 4:** Correlation analysis

			*BRCA1* ^*mut*^					sporadic				
			VDR	RXR	PPARγ	TRα	TRβ	VDR	RXR	PPARγ	TRα	TRβ
Spearman's rho	VDR	cc	1.000	0.336	0.538	–.008	.191	1.000	.042	0.256	.028	.097
		Sig.	x	.042*	.005*	.964	.257	x	.718	.024*	.810	.397
		n	37	37	25	37	37	78	78	78	78	78
	RXR	cc		1.000	0.578	–.172	0.395		1.000	–0.325	–.031	.045
		Sig.		x	.002*	.302	.014*		x	.003*	.786	.693
		n		38	26	38	38		79	79	79	79
	PPARγ	cc			1.000	–.001	.241			1.000	–.063	0.237
		Sig.			x	.997	.236			x	.582	.035*
		n			26	26	26			79	79	79
	TRα	cc				1.000	–.192				1.000	.195
		Sig.				x	.248				x	.071
		n				38	38				86	86
	TRβ	cc					1.000					1.000
		Sig.					x					x
		n					38					86

Correlation analysis was performed in *BRCA1* ^*mut*^ as well as in sporadic cases. Significant correlations are indicated by asterisks (*: *p <* 0.05)

### Absence of VDR or RXR is associated with reduced overall survival in *BRCA1*^*mut*^ cases

**Fig. 4:**
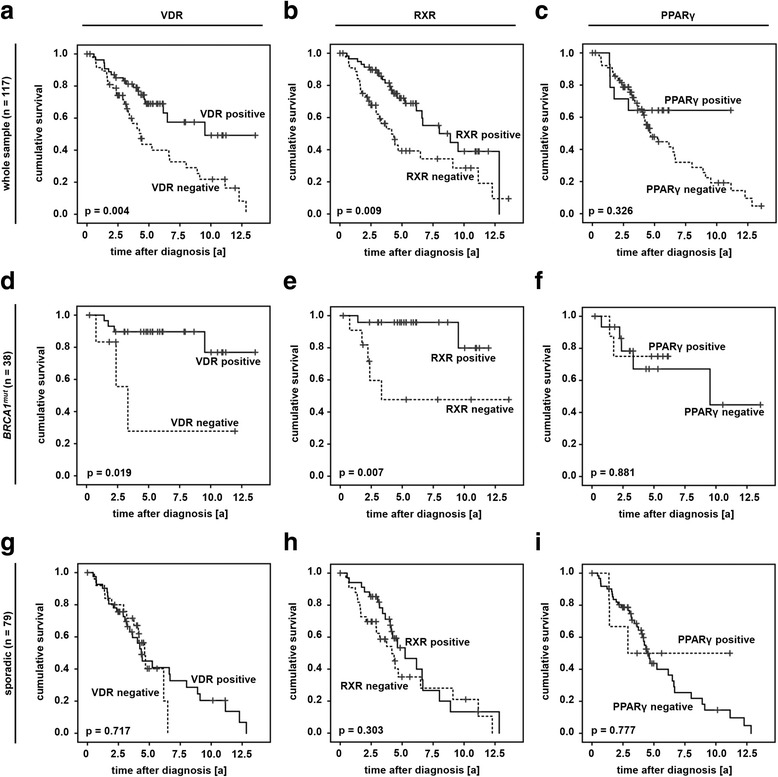
Immunopositivity of VDR and RXR predict favorable overall survival in *BRCA1* ^*mut*^ breast cancer. Survival analysis was performed on the whole patient sample (n = 117, a-c) as well as on BRCA1^mut^ (n = 38, d-f) and sporadic (n = 79, g-i) cancer cases

VDR, RXR and PPARγ were correlated to patients’ overall survival (OS). Studying the whole patient sample OS of those cases that stained negative for either VDR (95% CI (VDR negative): 4.43 y - 7.14 y; 95% CI (VDR positive): 7.39 y - 10.7 y; *p =* 0.004, Fig. 4a) or RXR (95% CI (RXR negative): 4.37 y - 7.47 y; 95% CI (RXR positive): 6.90 y - 9.72 y; *p =* 0.009, Fig. 4b) was found to be significantly reduced. However, no statistical association to OS was detected in case of sporadic cancer cases (Fig. 4g-i). Negativity for either VDR (95% CI (VDR negative): 0.69 y - 9.38 y; 95% CI (VDR positive): 10.3 y - 13.4 y; *p =* 0.019, Fig. 4d) or RXR (95% CI (RXR negative): 3.92 y - 11.3 y; 95% CI (RXR positive): 10.1 y - 12.2 y; *p =* 0.007, Fig. 4e) was associated with significantly shortened overall survival in those cases that had been diagnosed a *BRCA1* germline mutation. PPARγ was not related to OS at all (Fig 4c, f, i).

**Fig. 5:**
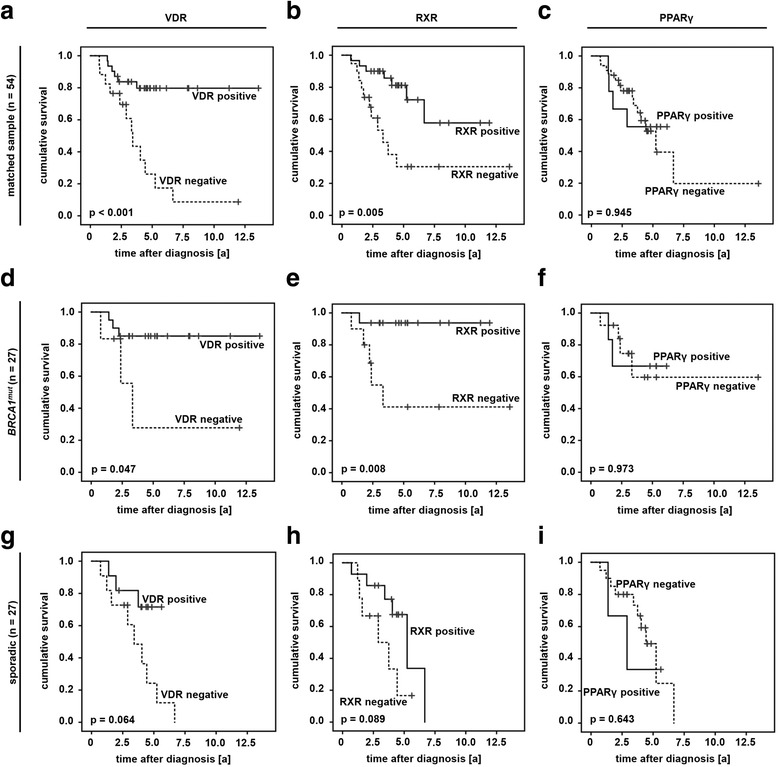
Immunopositivity of VDR and RXR predict favorable overall survival in *BRCA1* ^*mut*^ breast cancer. Survival analysis was performed on the matched patient sample (n = 54, a-c) as well as on BRCA1^mut^ (n = 27﻿, d-f) and sporadic (n = 27, g-i) cancer cases of the matched sample

Thus to avoid confounding effects survival analysis were repeated in those patients that had been matched for clinico-pathological variables. Interestingly, both VDR (95% CI (VDR negative): 2.47 y - 5.67 y; 95% CI (VDR positive): 9.61 y - 12.9 y; *p <* 0.001, Fig. 5a) and RXR (95% CI (RXR negative): 3.23 y - 8.52 y; 95% CI (RXR positive): 6.80 y - 10.8 y; *p =* 0.005, Fig. 5b) positivity predicted favorable OS in the matched patient panel prior to stratification. Following stratification this only remained to be significant in those patients that had been diagnosed a germline mutation in *BRCA1* (95% CI (VDR negative): 0.69 y - 9.38 y; 95% CI (VDR positive): 9.98 y - 13.7 y; *p =* 0.047, Fig. 5d; 95% CI (RXR negative): 2.98 y - 10.8 y; 95% CI (RXR positive): 10.1 y - 12.6 y; *p =* 0.008, Fig. 5e). Again, no association of PPARγ and OS was observed (Fig. 5c, f, i).

## Discussion

### VDR, RXR and PPARγ overexpression in *BRCA1*^*mut*^ breast cancer

To the best of our knowledge this analysis is among the first to show immunopositivity of VDR, RXR and PPARγ in *BRCA1* ^*mut*^ breast cancer patients. There is some evidence that absence of functional BRCA may alter expression of these so called ‘thyroid hormone receptor like genes’. For instance, knockdown of *BRCA2* in breast cancer cell lines modulated expression of RXR isoforms in opposing ways and knockout of *BRCA1* reduced expression of PPARγ in cardiomyocytes [24, 25]. Some authors even reported a physical interaction of VDR and BRCA1 protein [26, 27]. Hence, loss of functional *BRCA1* may lead to compensatory up-regulation of VDR. Whether a scenario like this may explain overexpression of VDR in *BRCA1* ^*mut*^ breast cancer cases remains to be elucidated. E3 Ubiquitin ligase activity of functional BRCA1 may serve as an alternative way to interpret overexpression of thyroid like receptors in a *BRCA1* mutant genetic background. Wildtype BRCA1 protein contributes to degradation of nuclear hormone receptors including VDR, RXR and PPARγ via its ubiquitinilation and sumoylation activity [28–31]. Thus loss of functional *BRCA1* may explain overexpression of these receptors in *BRCA1* mutant genetic background. A similar mechanism was demonstrated in case of thyroid hormone receptors in *BRCA1* ^*mut*^ breast cancer [8]. Besides carrying a *BRCA* mutation, cancers expressing alternative nuclear hormone receptors were often found to be triple negative. VDR, RXR and PPARγ were even detected in those cancers characterized as both triple negative and highly proliferative - two basic features of basal like breast cancer [22, 23]. Comprehensive analysis of “basal like biomarkers” performed in a larger patient sample is mandatory thus to properly select basal like breast cancer cases and to further study the role of alternative hormone receptors in basal like breast cancer.

### VDR and RXR in *BRCA1*^*mut*^ breast cancer - translational aspects

Though several significant associations of VDR, RXR and PPARγ to clinico-pathological variables were observed in sporadic breast cancer, there was only one significant correlation detected when VDR, RXR and PPARγ were correlated to clinico-pathological variables in *BRCA1* ^*mut*^ cases. A comprehensive study on VDR in 1116 breast cancer patients revealed VDR to correlate to those clinic-pathological variables that may indicate lower tumor-biologic aggressiveness [32]. We found that presence of VDR is positively correlated to the absence of distant metastasis. In line with others [15, 33] this suggests that VDR expression may exert differentiating effects on breast cancer cells. This is in agreement with the fact that absence of VDR was correlated with shortened OS of patients carrying a *BRCA1* mutation in the current analysis. However results on whether VDR may predict prognosis in breast cancer are not consistent throughout the literature. While a former study of our group detected VDR to correlate with favorable OS [16], others did not find an association of VDR and breast cancer prognosis [32]. So far, no data on the prognostic significance of VDR in *BRCA1* mutated breast cancer have been published. Whether the association of VDR and OS observed in the current work may be enhanced due to the absence of wildtype *BRCA1* remains to be validated in larger trials as well as on a functional level. Strikingly, Thakkar et al. recently highlighted that VDR agonists may inhibit proliferation of triple negative, VDR positive breast cancer cell lines in a receptor dependent manner [34]. This observation further supports a tumor suppressor like activity of VDR and may further strengthen a potential role of VDR as a promising new target in triple negative breast cancer.

There is some evidence that activation of RXR may induce apoptosis in breast cancer cells and may reduce cell growth [35, 36]. Even less is known on how RXR may affect tumorbiologic characteristics on *BRCA1* ^*mut*^ breast cancer and no data on prognostic significance of RXR in hereditary breast cancer have been published so far. Our results presented suggest that RXR positivity may predict favorable prognosis in breast cancer. Whether *BRCA1* ^*mut*^ breast cancer may be sensitive to RXR modulating drugs and whether this may affect tumor biology or even clinical outcome remains to be determined - with the same applying for VDR, respectively.

## Conclusion

The present study demonstrated *BRCA1* ^*mut*^ breast cancer cases to overexpress VDR, RXR and PPARγ - especially in the absence of ‘classical’ hormone receptors. Further loss of both VDR and RXR predicted shortened overall survival in *BRCA1* ^*mut*^ breast cancer. Therefore VDR may act as a tumor suppressor in presence of *BRCA1* ^*mut*^ and may potentially evolve as a promising new target in the future.

## Additional file

Additional file 1: Figure S1. Highly proliferative cancers within TNBC. (JPG 150 kb)

## Abbreviations

FFPE: Formalin fixed paraffin embedded
IRS: Immuno-reactive score
ns: Not significant
PPARγ: Peroxisome Proliferator-activated Receptor γ
RXR: Retinoid X Receptor
TRs: Thyroid hormone receptors
VDR: Vitamin D Receptor
