# Supplementary figures and images for: Vitamin D receptor, Retinoid X receptor and peroxisome proliferator-activated receptor γ are overexpressed in BRCA1 mutated breast cancer and predict prognosis
Source: J Exp Clin Cancer Res. 2017 Apr 20;36:57. doi: 10.1186/s13046-017-0517-1 (PMC5399435; doi:10.1186/s13046-017-0517-1)

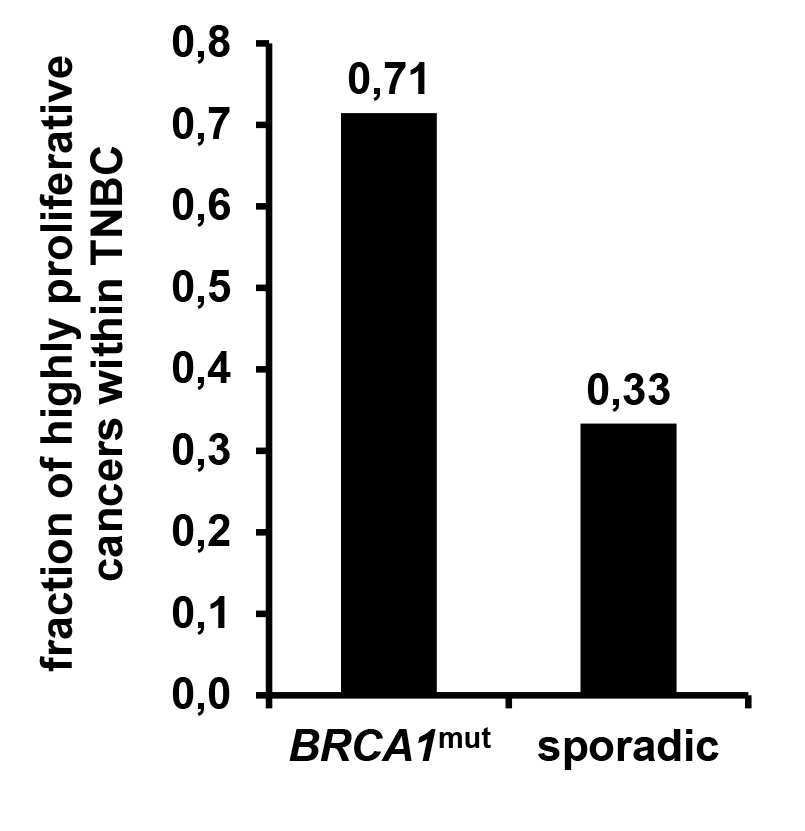

Supplement: Additional file 1: Figure S1. — Highly proliferative cancers within TNBC. (JPG 150 kb) [file 13046_2017_517_MOESM1_ESM.jpg]
